# AlOOH-Coated Glass Fiber-Reinforced Composites for Pipeline Rehabilitation: Enhancement of Interfacial Adhesion and Durability

**DOI:** 10.3390/ma18214887

**Published:** 2025-10-24

**Authors:** Mengfei Du, Xilai Yan, Chuandong Wu, Ke Wang

**Affiliations:** 1 National Engineering Research Center for Safe Sludge Disposal and Resource Recovery, Harbin Institute of Technology, Harbin 150090, China; 20b929012@stu.hit.edu.cn (M.D.);; 2 State Key Laboratory of Urban Water Resource and Environment, Harbin Institute of Technology, Harbin 150090, China; 3 Harbin Institute of Technology National Engineering Research Center of Water Resources Co., Ltd., Harbin Institute of Technology, Harbin 150090, China; 4 Guangdong Yuehai Water Investment Co., Ltd., Shenzhen 518021, China

**Keywords:** glass fiber-reinforced composites, unsaturated polyester resin, nano-AlOOH, sol-gel method, interfacial adhesion, mechanical property

## Abstract

Glass fiber (GF) reinforced unsaturated polyester resin (UP) composites are used in cured-in-place pipe (CIPP) rehabilitation technology of drainage systems due to their low cost and excellent force chemical properties. However, the weak interfacial compatibility between GF and the polymer matrix limits the stress transfer efficiency. Herein, a strategy of a polyhydric boehmite (AlOOH) layer coated on GF (GF-AlOOH) was developed for improving the mechanical properties of UP composites, and the enhancement effects of the coating process were analyzed. The AlOOH-modified GFs significantly improved the flexural and tensile strengths of the modified composites by 41.21% and 21.05%, respectively. Moreover, the enhancement mechanism was explored by analyzing the surface chemical structure of GF-AlOOHs. The nano-AlOOH was grafted on the GF surface by O=Al–OH. Meanwhile, the increase in the mechanical properties of UP/GF-AlOOH was mainly attributed to the combined effect of mechanical interlocking interaction, covalent bonding and hydrogen bonding, which improved the interfacial adhesion between GF and UP. In summary, this work provides effective guidance for achieving high-quality interfaces in GF composites and offers important insights into designing durable and cost-effective materials for CIPP rehabilitation and broader infrastructure applications.

## 1. Introduction

Unsaturated polyester resin (UP) used in glass fiber (GF) reinforced composites has excellent mechanical properties and outstanding chemical corrosion resistance and electrical properties, which makes it suitable for architecture fields, hydraulic engineering, boats, corrosion protection and electrical industry [1,2,3,4,5]. In addition, UP is among the most commonly used resins in the ultraviolet-cured-in-place pipe (UV-CIPP) rehabilitation technology of drainage systems [6,7,8,9]. The CIPP needs to replace the original pipe to bear the external pressure from the overburden and the internal pressure from the effluent inside the pipe. In order to ensure the safety and stability of the pipeline during use, CIPP materials have always pursued high strength. As a cost-effective reinforcing material, GF can significantly improve the strength, acid and alkali resistance and reduce the expansion coefficient of CIPP [6]. Moreover, GF has good flexibility and light transmittance. The composite hose formed by resin impregnated with GF can be adapted to various shapes of leaking pipes and fit closely to the inner wall of the pipe under pressure. After curing by UV irradiation, the resin and glass fiber form a hard and smooth lined pipe, thus realizing the repair and reinforcement of the pipe [10]. In general, the excellent properties of glass fiber-reinforced resin composites depend largely on their good interfacial adhesion [11,12,13]. However, the smoothness of the GF surface leads to poor interfacial adhesion between the fibers and the matrix, and stress concentration occurs easily [14]. Once the composite material is subjected to external forces, the load is difficult to transfer effectively from the matrix to the fibers [15].

In recent years, plasma treatments, acid-base etching and grafting coupling agents have commonly been used to modify GF surface to improve the bonding ability between resin and fibers [13,16,17]. However, it is difficult to control the etching effect of alkali on the GFs, resulting in a significant reduction in the strength of the GFs [14]. The effect of plasma treatment is non-permanent, and the aging process after treatment leads to a significant decrease in the wettability of the treated surface [18]. It is worth noting that chemical grafting can introduce rich polar groups through covalent bonds, thus significantly increasing the surface activity of GFs [17,19]. Adding nanomaterials has commonly been used to enhance the mechanical properties of composites [20,21]. Therefore, chemical grafting of nanomaterials onto GFs could create a high-quality interface with unsaturated polyester, which facilitates the formation of high-quality UV-cured pipes.

Inorganic fillers are commonly used to improve the mechanical properties of unsaturated polyester resin composites. The addition of aluminum oxide nanoparticles can improve the performance of polymer composites, because of their characteristics of high hardness, high strength, good oxidation resistance and wear resistance and exhibited excellent properties in terms of thermodynamics, chemistry and mechanical performance [22]. It is worth mentioning that Al_2_O_3_ powder has also been used to strengthen composites in recent years [23,24]. For example, adding 10 wt.% Al_2_O_3_ particles to glass fiber-reinforced epoxy composites resulted in 33% and 78% increases in flexural strength and flexural modulus, respectively [25]. Ba et al. [26] illustrated a modified densified bamboo composite consisting of Al_2_O_3_ nanoparticles and coupling agent KH550, whose tensile strength and flexion strength were 237% and 198% higher than those of natural bamboo, respectively. The obtained Al_2_O_3_-densified bamboo exhibited a rough and porous surface. The addition of Al_2_O_3_ can effectively promote the dislocation proliferation and make the grain withstand a high level of plastic strain, thus reducing the stress concentration at the grain boundary [27]. However, the phase separation between inorganic particles and resin matrix after curing could limit the enhancement of mechanical properties [28]. In this context, the chemical modification of fibers by boehmite (AlOOH) was more promising to reduce the damage to mechanical properties relative to the physical addition of them into the UP matrix.

The sol–gel method is a liquid phase technique used to produce nano powder. The nano-oxide particles generated by this method are small in size, narrow in distribution and high in purity [29,30,31]. Moreover, the equipment required for this preparation method is simple, and the process is easy to control, making it one of the most promising approaches for preparing high-performance nano-AlOOH [32,33]_._ Mat Rozi et al. [34] grafted SiO_2_ onto oil palm fiber via an in-situ silica sol–gel process method to prepare reinforced polypropylene composites, which acquired a 4.3% enhancement of the Young’s modulus. Ma et al. [35] modified an epoxy (EP) matrix with a polyhydric SiO_2_ coating assistant to graft organophosphorus for glass fiber-reinforced composites, and the tensile strength and impact strength were increased by 35% and 60%, respectively. The nano-oxide coating significantly increases the number of –OH groups, which facilitates reactions with coupling agents and enhances interface adhesion [36]. Based on the above advantages, it could be practical to make use of the sol–gel technique to prepare nano aluminum oxide for grafting GFs.

In this study, nano-AlOOH was grown in situ onto GFs using aluminum isopropoxide or aluminum nitrate as precursors under three reaction conditions. The physical and chemical properties of the modified GFs and the mechanical performance of the AlOOH-decorated glass fiber-reinforced unsaturated polyester resin composites (UP/GF-A) were comprehensively measured. In addition, the strengthening mechanism of UP/GF-A formed by three preparation methods of nano-AlOOH was also discussed. We believe that the strategies demonstrated in this work have improved thermal and mechanical properties for guiding the design and construction of composite materials, which can effectively promote their application in UV-curable pipeline materials.

## 2. Materials and Methods

### 2.1. Materials

The GF combination mat (Product Number: MK(ECP)300/600-960), with a density of 2.53 g/cm^3^ and thickness of 0.8mm per layer, was bought from Chongqing International Co., Ltd. (Chongqing, China). And the GF combination mat was composed of plain-woven roving cloth and chopped strand mat. The plain-woven roving cloth was stitched in biaxial fabric. The UP used was meta-Phenylneopentyl glycol (Palatal^®^ P 92 I-909), purchased from Jinling Aliancys Resin Co., Ltd. (Nanjing, China).

The additives were photoinitiator (1173, RYOJI), silane coupling agent (KH-570, ≥97%, Nanjing Chuangshi Chemical Auxiliary Co., Nanjing, China) and thermal initiation (benzoil peroxide, BPO, Dongguan Yunze New Material Technology Co., (Dongguan, China). Aluminum isopropoxide, aluminum nitrate hydrate (Al(NO_3_)_3_·9H_2_O) and isopropyl alcohol were purchased from Shanghai Aladdin Biochemical Technology Co. Ltd. (Shanghai, China). Nitric acid, acetyl acetone, sodium hydroxide and citric acid were purchased from Tianjin Zhiyuan Chemical Reagent Co. (Tianjin, China).

### 2.2. Synthesis of Glass Fiber Coated with AlOOH (GF-AlOOH)

**Figure 1:**
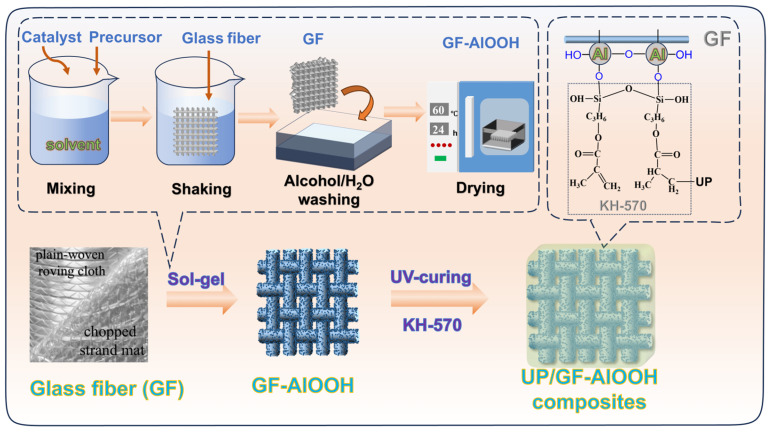
Schematic illustration of glass fibers grafted with polyhydric nano-AlOOH and composites preparation.

As shown in Figure 1, three sol-gel routes were developed to prepare AlOOH coatings on GFs by adjusting precursor compositions and reaction parameters. Aluminum isopropoxide, Al(NO_3_)_3_·9H_2_O, and citric acid with Al(NO_3_)_3_·9H_2_O were used as the main precursors to obtain GF-AlOOH-1 (GF-A1), GF-AlOOH-2 (GF-A2) and GF-AlOOH-3 (GF-A3), respectively. The specific differences among the three routes are summarized in Table 1. And the detailed preparation procedures are provided in the Text S1. Three specimens of the modified GF were prepared in each batch for subsequent testing.

### 2.3. Preparation of UP/GF Composites

The UP/GF prepreg were produced using the vacuum perfusion method. The UP was evenly mixed with photoinitiator 1173, BPO, KH-570 and defoamer (the mass ratio is 100:3:2:1). Then, the resin was pumped into the vacuum pumping device (XPR6910013, Zhuozhou, China) for 6 min to remove the bubbles. The prepreg was obtained after the resin was pumped into the vacuum bag to mix with five layers of GFs completely. Finally, the composites were cured under a 1000 W ultraviolet lamp positioned 30 cm above the surface for 6 min. Three specimens of the composite were prepared under identical conditions for the following tests. The fiber volume fraction of the UP/GF composites ranged from 44% to 48%, and the composite density was between 1.65 and 1.70 g/cm^3^. The specific values are presented in Table S1, with the calculation method described in Text S2 according to GB/T 2577-2005 [37].

### 2.4. Materials Characterization

The Fourier Transform Infrared spectra (PerkinElmer Frontier FTIR, Waltham, MA, USA) were used to detect chemical bonds and groups on the surface of GFs. The surface morphology of GFs was characterized using field emission scanning electron microscopy (FE-SEM, ZEISS SIGMA 500, Oberkochen, Germany). The elemental composition of the GF surfaces was analyzed by X-ray spectroscopy (EDS). The chemical composition of GF surfaces was observed using X-ray photoelectron spectroscopy (XPS, Thermo ESCALAB 250XI, Waltham, MA, USA). Atomic Force Microscopy (AFM, Bruker, Dimension Icon, Billerica, France) was used to characterize the physical morphology and roughness of GFs.

The wettability of the GFs was evaluated according to the contact angles (DCAT, θ) with two test liquids: water (surface tension *γ_l_* = 72.8 mN/m, dispersive component of surface tension $\gamma_{l}^{d}$ = 21.8 mN/m, polar component of surface tension $\gamma_{l}^{p}$ = 51.0 mN/m) and diiodomethane (surface tension *γ_l_* =50.8 mN/m, dispersive component of surface tension $\gamma_{l}^{d}$ = 48.5 mN/m, polar component of surface tension $\gamma_{l}^{p}$ = 2.3 mN/m) [38,39]. The optically determined angular contact apparatus (DataPhysics OCA25, Filderstadt, Germany) was used to measure the dynamic contact angles, and the surface energies were calculated using the WORK method. The surface energy (*γ_s_*) of the GFs was the sum of dispersion surface energy ($\gamma_{s}^{d}$*)* and polar surface energy ($\gamma_{s}^{p}$) as shown in Equation (1), where $\gamma_{s}^{d}$ and $\gamma_{s}^{p}$ are calculated by Equation (2).

(1) $$\gamma_{s}=\gamma_{s}^{d}+\gamma_{s}^{d}$$

(2) $$\gamma_{l}\left(1+\cos\theta \right)=2\sqrt{\gamma_{l}^{d}\gamma_{s}^{d}}+2\sqrt{\gamma_{l}^{p}\gamma_{s}^{p}}$$

The three-point short beam bending and tensile tests were performed using a universal electronic testing machine (Instron 3400, INSTRON^®^, Canton, OH, USA) according to GB/T 1449-2005 [40] and GB/T 1447-2005 [41] standards, respectively. The tests were conducted at room temperature with a crosshead speed of 2 mm/min. The bending specimens had dimensions of 80 × 15 × 4 mm^3^, while the tensile specimens measured 250 × 25 × 4 mm^3^. The thermal stability of UP/GF composites was determined using a thermogravimetric analyzer (TGA, NETZSCH STA449F5, Selbu, Germany) from 30 °C to 750 °C, with a heating rate of 10 °C/min in argon atmosphere.

## 3. Results and Discussion

### 3.1. Grafting Efficiency and Composition of GF-AlOOH

**Figure 2:**
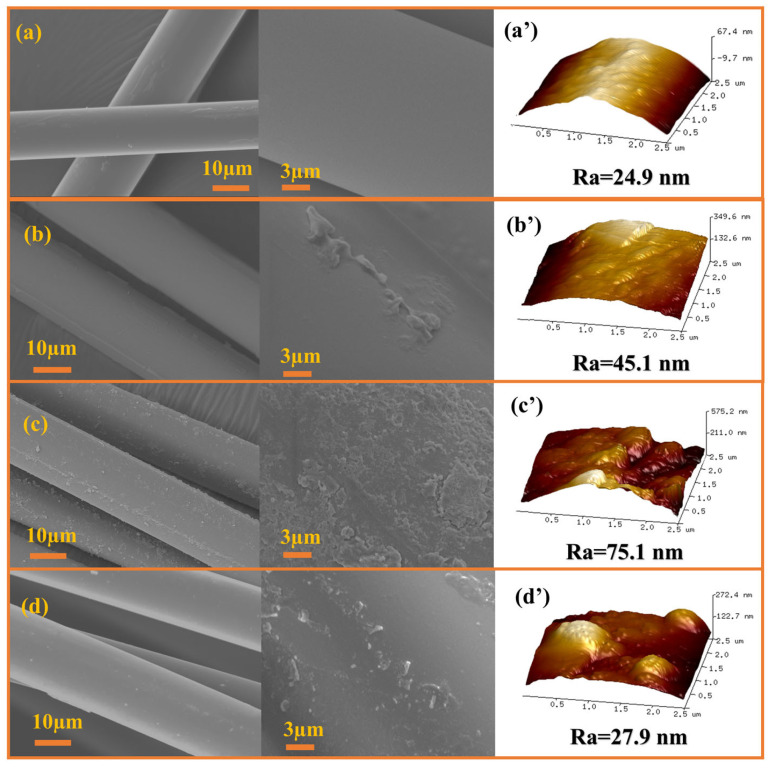
SEM and AFM images of different GF morphologies: (**a**,**a′**) untreated GF, (**b**,**b′**) GF-A1, (**c**,**c′**) GF-A2 and (**d**,**d′**) GF-A3.

The surface morphology and roughness of different GF samples was observed using SEM and AFM, respectively. The untreated GF surface (Figure 2a,a’) was smooth and the surface roughness (Ra) was only 24.9 nm. The untreated GF surface was smooth and exhibited only a few scratches. The roughness of all three GF-AlOOH samples was significantly improved. The AlOOH coated on the GF-A1 surface was formed using aluminum isopropoxide. The sol particles formed by this method were smaller than those when using Al(NO_3_)_3_·9H_2_O and easily formed a homogeneous colloid, so a stable coating layer could be formed, with an increased roughness of 45.1 nm (Figure 2b,b’). In comparison, GF-A2 (Figure 2c,c’) and GF-A3 (Figure 2d,d’) showed obvious rough particles on the surfaces, which could be a result of using Al(NO_3_)_3_·9H_2_O as the aluminum source. In particular, the maximum Ra was observed in GF-A2 (Ra = 75.1 nm), characterized by a dense distribution of nanoscale particles. Such topography effectively increased the contact area and mechanical interlocking at the interface, facilitating efficient stress transfer and suppressing debonding [42]. Citric acid was used as a complexing agent in the preparation of GF-A3, which prevented premature precipitation in subsequent steps, thus forming more dispersed AlOOH sol-gel aggregates. And the roughness of GF-A3 was lower than that of GF-A2, which was only 27.9 nm. In summary, using Al(NO_3_)_3_ as a precursor in combination with the aging process, resulted in the creation of rougher coating surface and effective contact area. This phenomenon not only improved physical interlocking but also facilitated the formation of covalent and hydrogen bonds with the UP matrix [43]. Therefore, the sol-gel parameters directly determined the surface morphology of GF-AlOOH, thereby controlling the efficiency of stress transfer across the GF/UP interface.

**Figure 3:**
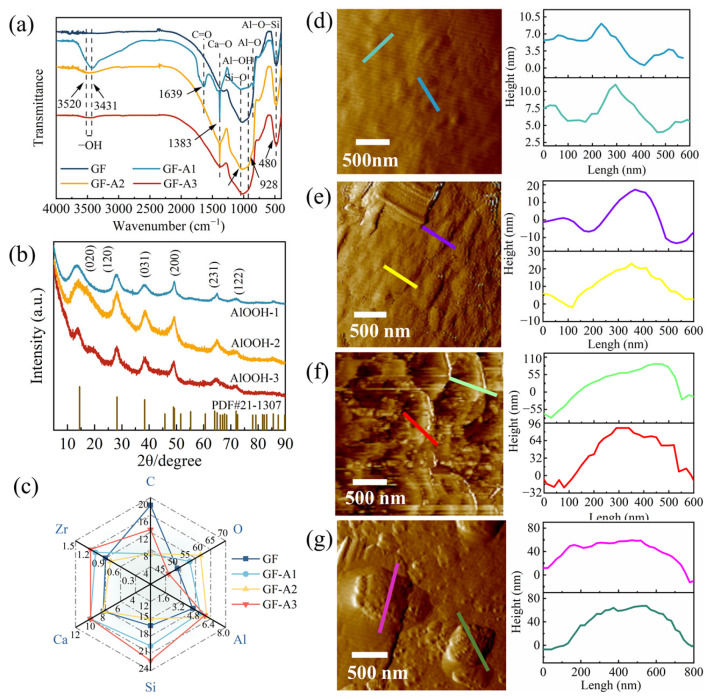
(**a**) XRD spectrum of AlOOH samples. (**b**) FTIR spectrum and (**c**) EDS results of GF samples. The corresponding height profiles of (**d**) untreated GF, (**e**) GF-A1, (**f**) GF-A2 and (**g**) GF-A3.

The XRD patterns of AlOOH powders prepared via three sol-gel routes are shown in Figure 3a. All samples exhibited characteristic diffraction peaks corresponding to γ-AlOOH (JCPDS PDF#21-1307), confirming that the desired boehmite phase was successfully obtained. However, notable differences were observed in peak intensity and width among the samples, reflecting variations in crystallinity and crystal growth behavior. The AlOOH-2 displayed relatively sharp and intense peaks, indicating the formation of well-crystallized γ-AlOOH. In contrast, the diffraction peaks of AlOOH-3 were significantly weaker, suggesting partial structural disorder. The peaks of AlOOH-1 showed intermediate characteristics, corresponding to a moderate degree of crystallinity. Such differences could be attributed to variations in hydrolysis and condensation rates during the sol-gel processes. A more balanced hydrolysis-condensation condition in GF-A2 likely facilitated ordered growth of γ-AlOOH crystallites. Based on the Debye-Scherrer equation, the average crystallite sizes of AlOOH-1, AlOOH-2 and AlOOH-3 were calculated to be 4.61, 3.40 and 4.20 nm, respectively, indicating that all samples consisted of ultrafine nano-crystallites. The nanoscale dimensions were favorable for forming uniform coatings on glass fiber surfaces, thereby enhancing resin wettability and interfacial adhesion. Moreover, the improved crystallinity of AlOOH-2 suggested enhanced structural stability, which may benefit its performance in composite applications.

The chemical composition and structure of GFs in different modification stages were characterized by FTIR spectroscopy. As shown in Figure 3b, the untreated GF displayed three absorption peaks at 480, 1017 and 1383 cm^−1^, attributed to the presence of Al–O, Si–O and Ca–O bonds on the original surface, respectively. All three GF–AlOOH samples showed peaks from 3431 to 3520 cm^–1^, which proved that the amount of –OH group significantly increased. This enhancement of –OH vibration bands indicated that the sol-gel coating process effectively introduced abundant hydroxyl groups, which could serve as active sites for chemical bonding with the UP resin. In case of GF-A1, the strong peak at 1639 cm^−1^ was attributed to the C=O stretching vibration of residual acetylacetone from the precursor gel. Additionally, GF-A1 displayed an abnormally strong and broad –OH band (3431 cm^−1^) with a distinct Al–O peak (~850 cm^−1^) but a weak Al–O–Si feature (~480 cm^−1^) [44]. Combined with its non-uniform coating (Figure 2b) and low crystallinity (Figure 3a), this suggested the presence of loosely aggregated physiosorbed aluminum-rich phases forming extensive hydrogen-bonding networks but limited covalent anchoring (Si–O–Al). In contrast, GF-A2 and GF-A3 exhibited stronger covalent integration of AlOOH layer with the GF surface. This balanced interfacial structure with ordered hydrogen bonding contributed to higher potential to enhance interfacial stability and mechanical properties.

The elemental composition of the GFs was analyzed using EDS tests. The untreated GF surface consisted of C, O, Si, Al, Ca and Zr (Figure 3c). The pristine GF exhibited a relatively high C content (18.2% At%), originating from the surface sizing agents. After nano-AlOOH was coated onto the GF surface, the elemental contents of O and Al in GF-AlOOH increased, with O increasing from 50.7% to 60.0% and Al from 4.5% to 6.0%, confirming that the successful deposition. The increase in O and Al indicated the formation of hydroxyl-rich sites on the GF surface, which might enhance interaction with coupling agents. Meanwhile, the C signal was markedly reduced, particularly for GF-A2 and GF-A1. These indicated that the effective removal of organic residues and exposure of a cleaner surface. Among the modified samples, GF-A2 displayed a pronounced enhancement in Al content accompanied by a significant reduction in C, suggesting the formation of a uniform and well-adhered AlOOH layer. GF-A1 and GF-A3 also showed higher Al signals but were accompanied by a more drastic decrease in Si, implying a thicker and potentially less homogeneous coating. The sol-gel route used for GF-A2 provided the most effective AlOOH coverage, balancing sufficient deposition with uniformity, which was expected to improve the interfacial compatibility between the GFs and the resin matrix. The EDS results are consistent with the SEM and FTIR analyses described above.

The morphology of the GFs coated with AlOOH was characterized by AFM and the results are shown in Figure 3d–g. The surface of the untreated GF was smooth (Figure 3d), and the surface scratches were about 2.5 nm deep after ethanol elution process. The height profile of the GF-A1 surface along the horizontal line (Figure 3e) demonstrated that the thickness of the AlOOH coating layer was about 20 nm. Typically, the AlOOH particles attached to GF-A2 were clearly clustered and tightly packed, with a height of about 120 nm (Figure 3f). Moreover, the nano-AlOOH coated onto GF-A3 was flat (Figure 3g), with a height of about 65 nm and a width of up to 600 nm. These distinct surface morphologies indicated that the sol-gel parameters strongly influenced the nucleation and growth of AlOOH. In particular, the densely packed nanostructure on GF-A2 provided a larger effective surface area and stronger potential for mechanical interlocking with the resin matrix, which was expected to enhance interfacial adhesion.

**Figure 4:**
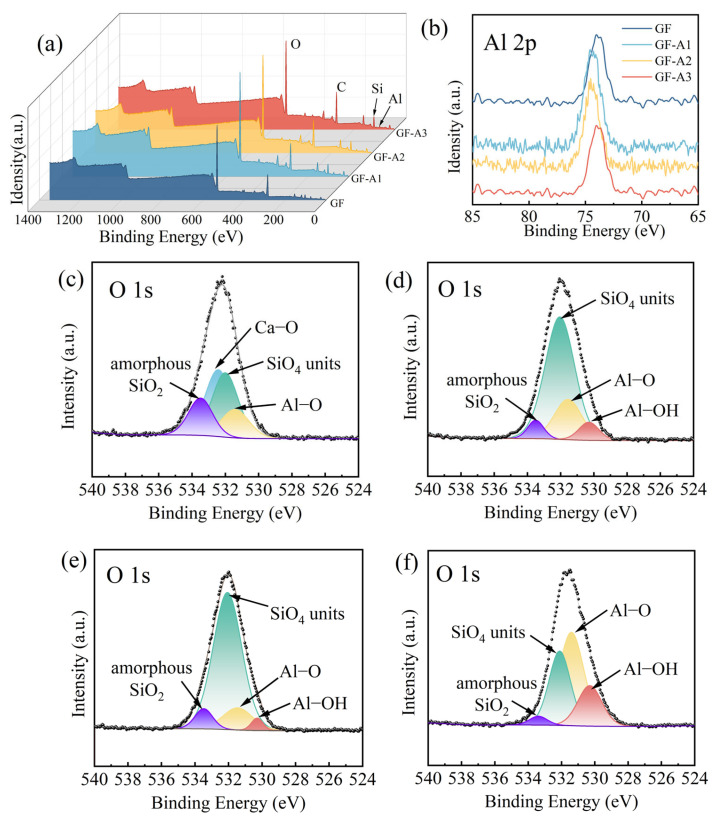
(**a**) Wide-scan XPS spectra and (**b**) Al2p spectra of GF samples. O1s spectra of (**c**) untreated GF, (**d**) GF-A1, (**e**) GF-A2 and (**f**) GF-A3. (The grey line: fitting curve; black circles: raw data.)

The chemical constitution of different modified GF surfaces was determined by XPS tests. The wide-scan XPS spectrum (Figure 4a) showed that the GF surfaces were chiefly composed of O, C, Si and Al. The increased peaks of Si elemental at 102.5 eV and Al elemental at 74.3 eV in the GF-AlOOH samples indicated the successful grafted of nano-AlOOH on the GF surfaces. The XPS spectra of Al2p core levels are shown in Figure 4b. The intensity peak of the untreated GF was around 73.98 eV, while the peak of GF-A1, GF-A2 and GF-A3 increased to 74.43, 74.53 and 74.03 eV, respectively. These shifts, along with the characteristic separation into Al–OH (74.8 eV) and Al–O (74.10 eV) components, indicated that the GF--AlOOH samples contained a higher proportion of Al–OH. These proved that the Al element was grafted onto GFs as γ-AlOOH via O=Al–OH. Furthermore, the shift in the Al 2p binding energy toward higher values for GF-A1 and GF-A2 suggested stronger chemical interactions between AlOOH and the surface silanol groups of glass fiber. Such bonding implied the formation of stable Al–O–Si linkages at the interface.

The O1s high-resolution spectrum of different GFs are exhibited in Figure 4c–f. As shown in Figure 4c, there were four main characteristic peaks of untreated GF at 533.5, 532.4, 532.0 and 531.5 eV, corresponding to bridging oxygen atoms in amorphous SiO_2_, Ca–O, nonbridging oxygens of SiO_4_ units and Al–O, respectively [45,46]. After grafting nano-AlOOH, a new peak at 530.3 eV appeared in the GF-AlOOH samples (Figure 4d–f), representing Al–OH groups (Figure 4d–f) [47]. Some of these –OHs could form hydrogen bonds with the oxygen atom of UP chains. Notably, the content of SiO_4_ units was the highest in GF-A2 than the other modified GFs. NaOH was used as a catalyst in the preparation of GF-A2. The polycondensation reaction was accelerated under alkaline conditions, and it is easy to form a large molecular dry gel with a loose structure, thus leading to a higher Si–O exposure. The above results indicated that the nano-AlOOH were grafted onto the GF by covalent and ionic bonding together. The XPS results were complemented by FTIR spectroscopy.

### 3.2. Surfaces Wettability and Energetics of GF-AlOOH

**Figure 5:**
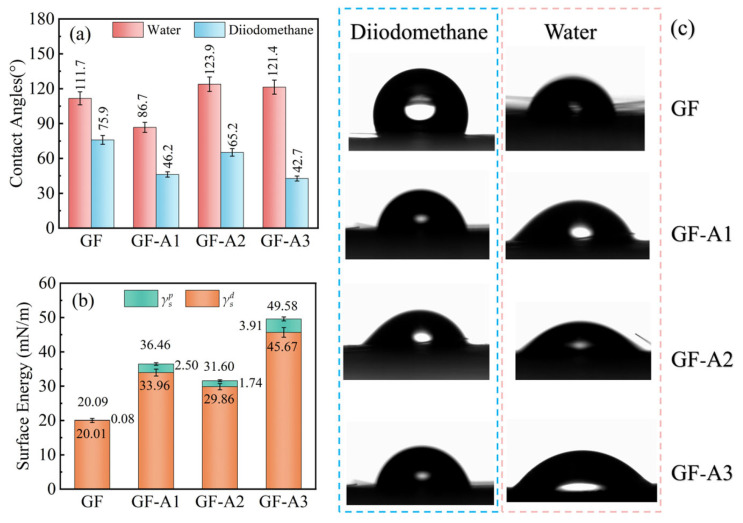
(**a**) Contact angle and (**b**) surface energy of various GFs. (**c**) Contact angle images on the GF surfaces.

The surface energy of GFs can be calculated from the contact angles measured by the DCAT tests, which is closely related to the interfacial adhesion [48]. As shown in Figure 5a, the untreated GF had the largest water contact angle of 111.7°, indicating a strongly hydrophobic surface. After modification, the contact angles of all samples decreased, demonstrating a significant improvement in surface hydrophilicity. Among them, GF-A2 showed the lowest water contact angle of 68.0°. This suggests that the densely packed AlOOH nanostructure exposed abundant hydroxyl groups, which were expected to increase the surface polarity of GF and promote resin wetting. Moreover, a similar trend was observed for diiodomethane, further confirming the enhanced surface wettability after modification. Representative contact angle images are provided in Figure 5c, which visually illustrate the distinct differences in droplet shape.

The corresponding surface energies calculated from the contact angles are shown in Figure 5b. The unmodified GF had the minimum surface energy at 26.94 mN/m, dominated almost entirely by the dispersive component (26.91 mN/m), reflecting its nonpolar and chemically inert surface. The surface energy of GF-A1, GF-A2 and GF-A3 were 31.58, 42.54 and 39.07 mN/m, respectively. Furthermore, the dispersion component of GF-A2 was 31.35 mN/m and the polarity component was 11.19 mN/m, suggesting a substantial contribution from polar hydroxyl groups generated on the AlOOH coating [49]. Such a balanced increase in both components implied that the GF surface became more polar and energetically favorable for resin wetting and diffusion. The high surface energy was expected to facilitate the diffusion and impregnation of the resin on the GF surface, thereby enhancing the interfacial adhesion [48]. Overall, the positive correlation between Al–OH content and surface energy further confirmed that surface hydroxylation played a dominant role in improving wettability with the UP matrix.

### 3.3. Thermal Behavior of UP/GF-AlOOH Composites

**Figure 6:**
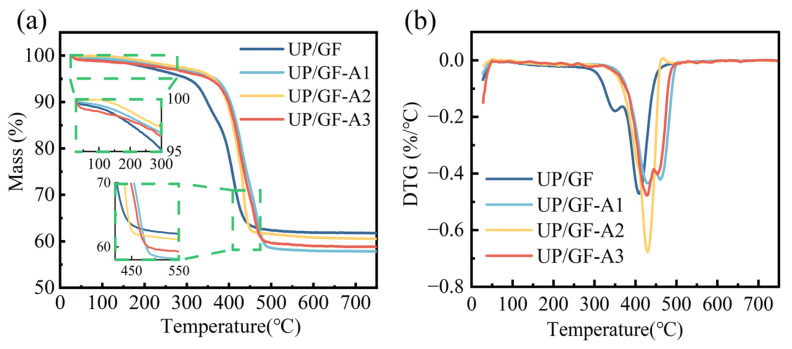
(**a**) TGA and (**b**) DTG curves of UP/GF composites.

The thermal stability of different UP/GF composites was investigated using the TGA results, as shown in Figure 6a. All UP/GF samples showed a slight mass loss at 28–48 °C due to the evaporation of bound water. The UP in the composites began to decompose from 120 °C and the mass loss reached 6.6% until 320 °C, attributed to the degradation of thermal stabilizers and other additives. Significant decomposition occurred between 320 °C and 430 °C, corresponding to the breakdown of the resin matrix (Figure S1b), with a final mass residue of 61.8% at 750 °C for UP/GF. The above thermal behavior of UP composites was in agreement with Laoubi et al. [50]. In the case of UP/GF-AlOOH samples, the mass loss until 370 °C was 4.9%, 5.2% and 5.9% for UP/GF-A1, UP/GF-A2 and UP/GF-A3, respectively. The thermal degradation process from 28 to 370 °C showed that nano-AlOOH-grafted composites improved the stability of the composites by reducing their weight loss and delaying the start temperature of the UPs’ decomposition. The strong chemical bonds such as Si–O–Al and C–C were formed between the AlOOH layer and the silane coupling agent at the GF/UP interface. Such a covalent bond network limited the mobility of UP chain segments adjacent the fiber surface,thereby delaying enhancing the thermal stability of the resin. [51]. At 750 °C, the residual masses of UP/GF-A1, UP/GF-A2 and UP/GF-A3 were 57.8%, 60.6% and 58.8%, respectively. A smaller residual mass corresponded to a higher amount of nano-AlOOH attached to the fiber surface. [48].

The DTG curves of UP/GF composites are shown in Figure 6b. All samples exhibited major degradation peaks between 350 and 500 °C, corresponding to the decomposition of the UP matrix. For the unmodified UP/GF composite, initial weight loss occurred near 300 °C, which is consistent with the decomposition of UP. A second inflection point appeared around 370 °C, corresponding to the thermal behavior of GF (Figure S1a). UP/GF-A3 showed a peak shift from 407 °C to 427 °C compared with the unmodified UP/GF, suggesting that the AlOOH coating strengthened interfacial bonding and restricted polymer chain mobility to improve thermal stability. Moreover, the sharpest and deepest peak with the highest mass-loss rate appeared in UP/GF-A2, indicating a larger amount of resin decomposition occurring within a narrow temperature window. This behavior was consistent with the SEM and XRD results, which confirmed higher surface coverage and crystallinity of AlOOH in UP/GF-A2, leading to stronger confinement of UP chains but also a more concentrated degradation process. In contrast, UP/GF-A3 displayed a broader and lower-intensity peak, suggesting that the coating was thinner, which slowed the resin degradation. AlOOH modification significantly enhanced the thermal stability of UP composites, with GF-A2 exhibiting optimal thermal stability due to its higher coating thickness and crystallinity.

### 3.4. Mechanical Properties of UP/GF-AlOOH Composites

**Figure 7:**
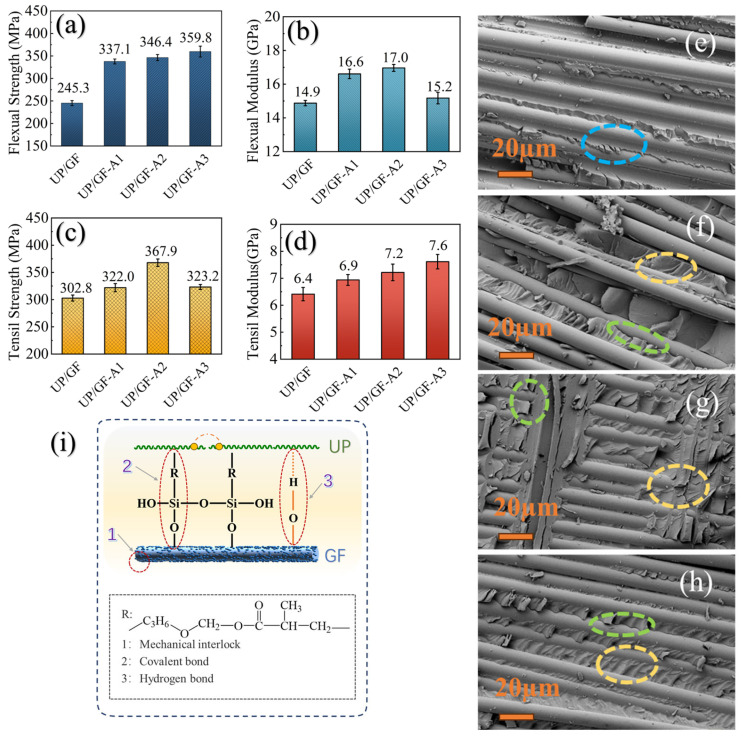
Mechanical properties of UP composites: tensile strength (**a**), tensile modulus (**b**), flexural strength (**c**) and flexural modulus (**d**). SEM images of fracture cross section after tensile test of the composites reinforced by (**e**) untreated GF, (**f**) GF-A1, (**g**) GF-A2 and (**h**) GF-A3. Schematic diagram of the possible interactions between GFs and UP chains (**i**). (Blue line: smooth displacement; green line: matrix cracking; yellow line: matrix fracture.)

The flexural strength and modulus and tensile strength and modulus of UP/GF composites were detected to further investigate the effects of different grafting treatments on mechanical properties. The results are shown in Figure 7a–d. All UP/GF-AlOOH composites exhibited higher strength and modulus than the unmodified UP/GF, indicating significant reinforcement by the nano-AlOOH coating. Notably, UP/GF-A2 had the highest flexural modulus (17.0 GPa) and tensile strength (367.9 MPa), which increased by 14.09% and 21.50% compared to UP/GF, respectively. In addition, UP/GF-A3 exhibited the highest flexural strength and tensile modulus, with increases of 46.68% and 18.75% compared to UP/GF. Based on the enhancement of these four mechanical properties, the order of these three grafting treatments was UP/GF-A2> UP/GF-A3> UP/GF-A1. As shown in Table S2, the design flexural strength and modulus required for CIPP systems are 45 MPa and 10,000 MPa, respectively. The UP/GF-AlOOH composites achieved flexural strength and modulus values of 359 MPa and 170,000 MPa, which far exceed these design requirements. In addition, the tensile strength of UP/GF-A2 was 6 and 18 times higher than the limits specified in the Chinese and international standards, respectively. Overall, the UP/GF-AlOOH composites demonstrated mechanical properties that surpass the strength and modulus requirements defined in ASTM F1216-22 [52], GB/T 41666.4-2024 [53] and T/CECS 559-2018 [54], underscoring their strong potential as CIPP liner materials for pipeline rehabilitation.

Considering the phase separation between GFs and UP after curing, the high mechanical strength of the composites was based on a sufficient bonding and interaction to ensure that the fibers could take up the loads from the resin matrix [35]. The possible interactions between GF-AlOOH and UP molecules were analyzed, in order to explore the enhancement mechanism of GF-AlOOH in the UP system. As shown in Figure 7i, the grafted nano-AlOOH might enhance the interfacial adhesion between GFd and UP through three bonding interactions. The fracture interface of UP/GF after tensile test (Figure 7e) displayed clear and smooth displacement between the resin matrix and the GFs (blue line), so neat fiber pullout was the primary cause of composite failure, indicating that adhesion damage was the damage mode of UP/GF.

Notably, the fractures of the resin matrix were rough and irregular for UP/GF-AlOOH (yellow line), and only a small amount of peeling appeared between the resin and the GF (green line) (Figure 7f–h). The pronounced surface roughness of UP/GF-A2 enhanced mechanical interlocking, allowing UP to fill the grooves on the fiber surface and ensuring close contact and anchoring between the GF and UP (yellow line). This could potentially change the failure mode of composites from adhesion failure to a combination of adhesive failure and cohesive failure [48,49]. Further, the higher wettability of the modified GF surface prevented the stress concentrations due to voids and defects at the interface between the GF and UP. The highest surface energy of UP/GF-A2 might reduce surface tension and polarity differences with UP, contributing to its highest flexural modulus and tensile strength. Additionally, UP/GF-A2 and UP/GF-A3 contained more Si–OH and Al–OH than UP/GF-A1 and thus could form more covalent bonds with kh-570 (green line). Covalent bonds were considered to be the strongest type of fiber-resin bonds according to chemical bonding theory [55]. During the curing process, the Si–OH groups of KH-570 undergo hydrolysis and react with the Al–OH groups on the surface of GF-AlOOH to form Si–O–Al bonds, while the C=C groups at the other end participate in the copolymerization with the resin matrix. The introduction of AlOOH and KH-570 establishes a reinforced chemical bonding network between GF and UP, thereby enhancing the interfacial adhesion strength. Chen et al. [56] revealed that small amounts of nano-oxides could significantly improve their mechanical properties through intermolecular hydrogen bonding and polymer chain shrinkage. Similarly, the hydroxyl bonds of nano-AlOOH had the potential to react directly with resin chains, which allowing the GFs to be firmly anchored in the cross-linked structure of the UP macromolecular network.

## 4. Conclusions

In conclusion, this study successfully developed a strategy to enhance the mechanical properties of UP/GF composites by coating polyhydric nano-AlOOH onto GFs. Among the three modified UP/GF composites, the GF-AlOOH prepared with Al(NO3)3·9H2O under alkaline conditions exhibited higher surface roughness, grafting amount, and surface energy. The synergistic reinforcement mechanism involved enhanced mechanical interlocking from the rough surface, abundant covalent bonding provided by the grafted nano-AlOOH and improved compatibility and hydrogen bonding due to increased surface energy. The insights and methodology establish a solid foundation for designing cost-effective, high-performance fiber-reinforced composites for industrial applications such as UV-CIPP and are anticipated to inspire further exploration of nanomaterial grafting for advanced composite materials.

## Figures and Tables

**Table 1:** Sol-gel preparation conditions for AlOOH-coated glass fibers.

Sample	Precursor	Solvent System	Reaction Condition	Catalyst
GF-A1	aluminum isopropoxide	Isopropanol	85–95 °C	nitric acid
GF-A2	Al(NO_3_)_3_·9H_2_O	H_2_O	room temperature	NaOH
GF-A3	Al(NO_3_)_3_·9H_2_O and citric acid	H_2_O	75 °C	ammonia solution

## Data Availability

The original contributions presented in this study are included in the article/Supplementary Materials. Further inquiries can be directed to the corresponding author.

## Supplementary Materials

The following supporting information can be downloaded at: https://www.mdpi.com/article/10.3390/ma18214887/s1, Text S1: Synthesis of glass fiber coated AlOOH (GF-AlOOH); Text S2: Test method for GF volume fraction in UP/GF composites; Table S1: Comparison of glass fiber volume fraction in composites; Figure S1: DTG curves of (a) original GF and (b) UP resin; Table S2: Comparison of Material Properties and Standard Requirements [52,53,54,57,58,59,60].

## Abbreviations

The following abbreviations are used in this manuscript:

GF	Glass fiber
UP	unsaturated polyester resin
UV–CIPP	Ultraviolet-cured-in-place pipe
AlOOH	Boehmite
BPO	Benzoil peroxide
