# Supplementary material for: AlOOH-Coated Glass Fiber-Reinforced Composites for Pipeline Rehabilitation: Enhancement of Interfacial Adhesion and Durability
Source: Materials (Basel). 2025 Oct 24;18(21):4887. doi: 10.3390/ma18214887 (PMC12608212; doi:10.3390/ma18214887)
Supplement: Supplementary file 1 [file materials-18-04887-s001.zip › materials-3923830-supplementary.pdf]

materials

MDPI

Supplementary Materials

AlOOH-Coated Glass Fiber-Reinforced Composites for Pipeline Rehabilitation: Enhancement of Interfacial Adhesion and Durability

Mengfei Du [1,2], Xilai Yan [1], Chuandong Wu [3,4] and Ke Wang [1,2,*]

1 National Engineering Research Center for Safe Sludge Disposal and Resource Recovery, Harbin Institute of Technology, Harbin 150090, China
2 State Key Laboratory of Urban Water Resource and Environment, Harbin Institute of Technology, Harbin 150090, China
3 Harbin Institute of Technology National Engineering Research Center of Water Resources Co., Ltd., Harbin Institute of Technology, Harbin 150090, China
4 Guangdong Yuehai Water Investment Co., Ltd., Shenzhen 518021, China
* Correspondence: 20110149@hit.edu.cn; Tel.: +86-451-86283001

Text S1 Synthesis of glass fiber coated AlOOH (GF-AlOOH)

S1.1 Synthesis method of GF-AlOOH-1 (GF-A1)

The aluminium isopropoxide (10 g) was dissolved in isopropanol (100 ml) to make the alcohol-aluminium phase in a water bath at a temperature of 85-95°C. Then 196 ml deionized water/ isopropanol solution (V/V=1:1), and 3 drops nitric acid were mixed to make the aqueous phase. The aqueous phase was added dropwise in the alcohol-aluminium phase while stirring at 400 rpm. Mechanical stirring of the reactor was required for adequately hydrolysis of aluminium isopropoxide and to increase the rate of hydrolysis [1]. A piece of GF (9 cm*9 cm) was added and aged for 4 h. The gel was washed three times with deionized water and ethanol, respectively. Then, the gel was dried at 80 °C to form a dry gel. Finally, the GF was dried under vacuum at 60 °C for 24h to produce the GF-A1 sample.

S1.2 Synthesis method of GF-AlOOH-2 (GF-A2)

18.76g $Al(NO_3)_3·9H_2O$ was dissolved in 50ml deionized water, and 1ml acetylacetone was added to the reactor, then stirred at 400 rpm for 10-20 minutes. Afterward, 150mL sodium hydroxide solution(NaOH, 1mol/L) was added drop by drop [2]. Typically, a piece of GF (9cm*9cm) was immersed in the reactor and reacted for 30min, after which it was washed three times with deionized water and ethanol. At last, the GF was put into vacuum to dry for 24h at the temperature of 60 °C to obtain the GF-A2 sample.

S1.3 Synthesis method of GF-AlOOH-3 (GF-A3)

24.2 g of $Al(NO_3)_3·9H_2O$ and 21.0 g of citric acid were dissolved in a reactor containing 150 mL of distilled water. The mixture was stirred at 300 rpm for 10 minutes until complete dissolution. Ammonia solution was added dropwise to the mixed solution, and the pH was adjusted to 8.5 using a calibrated digital pH meter. The reactor was maintained at 75°C in a water bath [3,4]. A piece of GF (9 cm*9 cm) was added to the sol, and the pH of the mixed solution was monitored every 30 min using pH indicator paper. If necessary, additional ammonia solution was added to maintain the pH around 8. When

the solution turned into a white viscous state, the mixture was further aged for 6 h. Then, the GF sample was washed three times with deionized water and anhydrous ethanol before drying in an 80 °C oven. The dry gel was washed with water and alcohol. Finally, the GF-A3 sample was obtained after vacuum drying at 60 °C for 24h.

Text S2 Test method for GF volume fraction in UP/GF composites

The volume fraction of glass fiber was calculated according to GB/T 2577-2005 in the steps shown below: A 2 g GFRP sample was initially placed in a desiccator for 24 h. The sample was then dried in an oven at 80 °C for 2 h and allowed to cool to room temperature within the desiccator.The pretreated specimen was transferred to a 50 mL crucible (m_1), heated at 625 °C for 10 min, and cooled to room temperature for weighing. This heating and weighing cycles were repeated until the difference between successive measurements did not exceed 1 mg. The crucible containing the specimen was weighed (m_2) before placing it into a muffle furnace. The temperature was first raised to 350 °C for 30 min and then increased to 625 °C until complete combustion of all carbon. The crucible with the remaining residue was removed from the furnace and weighed (m_3). The fiber volume fraction of UP/GF sample was determined using equation (A.1).

For other modified samples, the residues were transferred to a beaker containing 20 mL of hydrochloric acid and stirred until complete reaction. The crucible was rinsed multiple times with distilled water, and the rinses were combined with the beaker contents. The mixture of glass fibers and acid was then filtered through a dry filter and washed repeatedly with distilled water until neutral. The residues were subsequently washed three times with alcohol, dried to constant weight in an oven, and weighed (m_4). The fiber volume fraction of GFRP samples was calculated according to equation (A.2). The resulting data are summarized in Table S1.

Table S1. Comparison of glass fiber volume fraction in composites.

Samples	m_1 (mg)	m_2 (mg)	m_3 (mg)	m_4 (mg)	Volume (cm^3)	ρ_c (g/cm^3)	ρ_f (g/cm^3)	V_g (%)
UP/GF	51.5966	53.7174	53.0320	/	1.28	1.65	2.53	44.23
UP/GF-A1	49.9950	51.8522	52.2200	1.3194	1.12	1.66	2.53	46.70
UP/GF-A2	49.8867	51.8997	51.1303	1.4329	1.19	1.69	2.53	47.52
UIP/GF-A3	47.8372	49.9626	49.2045	1.4408	1.25	1.70	2.53	45.48

$$V_g = \left[\frac{m_3\text{-}m_1}{m_2\text{-}m_1} \times \frac{\rho_c}{\rho_f}\right] \times 100 \quad \text{(S. 1)}$$

$$V_g = \left[\frac{m_4}{m_2\text{-}m_1} \times \frac{\rho_c}{\rho_f}\right] \times 100 \quad \text{(S. 2)}$$

V_g: the fiber volume content in GFRP, %.

ρ_c: density of GFRP specimens, g/cm^3.

ρ_f: density of glass fiber, 2.53 g/cm^3.

m_2: total mass of crucible and specimen, mg.

m_3: total mass of crucible and residue after burning, mg.

m_1: crucible mass, mg.

m_4: mass of dried samples after pickling, mg.

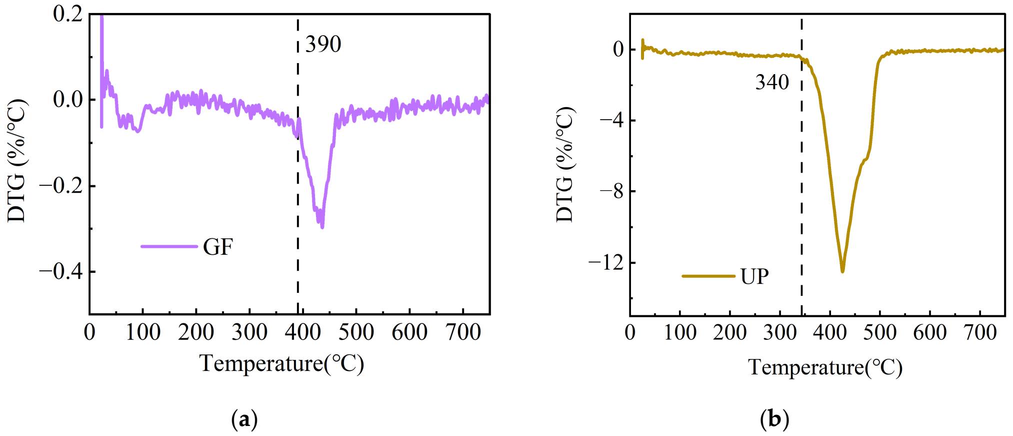

Figure S1. DTG curves of (**a**) original GF and (**b**) UP resin.

Table S2. Comparison of Material Properties and Standard Requirements.

Mechanical Characteristics	This Study				Chinese Standard [5,6]	ASTM F1216-22 [7]
	UP/GF	UP/GF-A1	UP/GF-A2	UP/GF-A3		
Flexural strength (MPa)	245	337	346	359	45	31
Flexural modulus (MPa)	14900	16000	17000	15200	10000	1724
Tensile strength (MPa)	302	322	368	323.2	62	21(for pressure pipes only)
Tensile modulus (MPa)	6400	6900	7200	7600	Not mentioned	Not mentioned

Reference

1. Huang, Y.L.; Xue, D.S.; Zhou, P.H.; Ma, Y.; Li, F.S. α-Fe–Al2O3 nanocomposites prepared by sol–gel method. *Mater. Sci. Eng. A* **2003**, *359*, 332–337. https://doi.org/10.1016/S0921-5093(03)00374-5.
2. Niero, D.F.; Montedo, O.R.K.; Bernardin, A.M. Synthesis and characterization of nano α-alumina by an inorganic sol–gel method. *Mater. Sci. Eng. B* **2022**, *280*, 115690. 10.1016/j.mseb.2022.115690.
3. Yabuki, M.; Takahashi, R.; Sato, S.; Sodesawa, T.; Ogura, K. Silica–alumina catalysts prepared in sol–gel process of TEOS with organic additives. *Phys. Chem. Chem. Phys*. **2002**, *4*, 4830–4837. https://doi.org/10.1039/b205645c
4. Suhasinee Behera, P.; Bhattacharyya, S.; Sarkar, R. Effect of citrate to nitrate ratio on the sol-gel synthesis of nanosized α-Al_2O_3 powder. *Ceram Int*. **2017**, *43*, 15221–15226. https://doi.org/10.1016/j.ceramint.2017.08.057
5. *GB/T 41666.4-2024*; Plastics piping systems for renovation of underground non-pressure drainage and sewerage networks-Part 4: Lining with cured-in-place pipes. Standards Press of China: Beijing, China, 2024.
6. *T/CECS 559-2018*; Specification for water and drainage pipeline rehabilitation using cured-in-place-pipe method. Planning Press: Beijing, China, 2018.
7. *ASTM F1216-22*; Standard Practice for Rehabilitation of Existing Pipelines and Conduits by the Inversion and Curing of a Resin-Impregnated Tube. ASTM: West Conshohocken, PA, USA, 2022.
